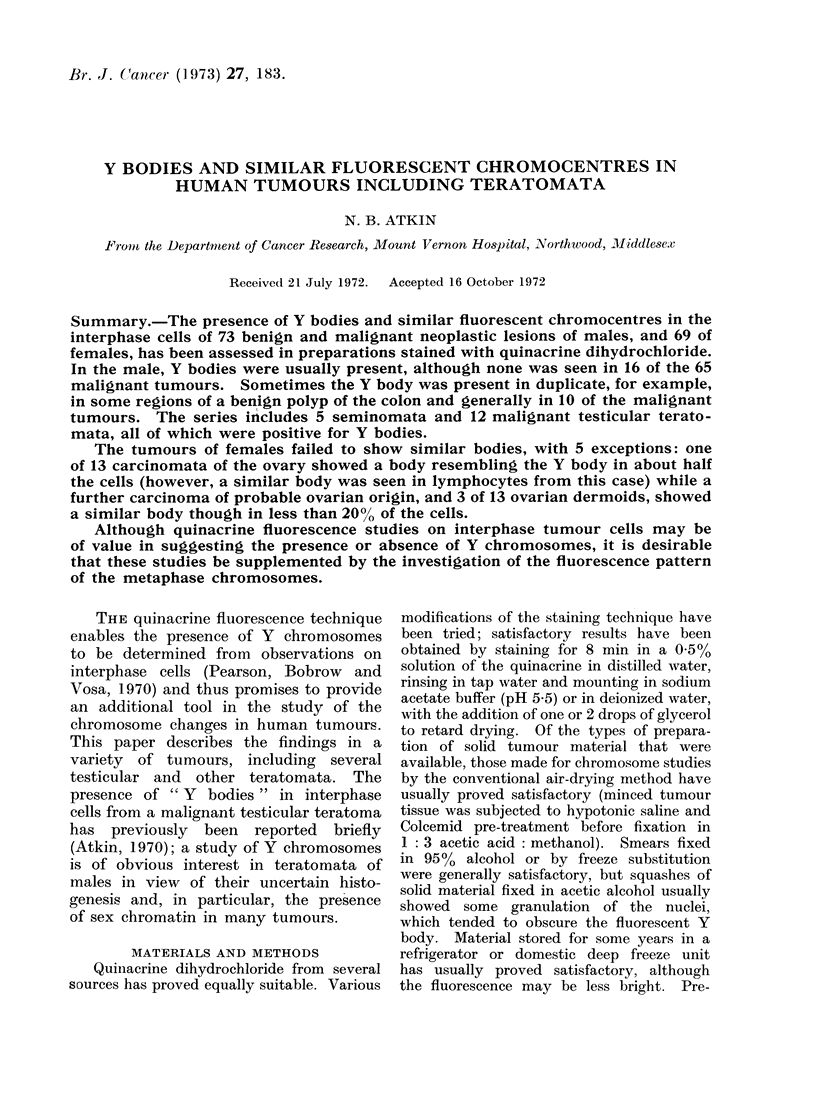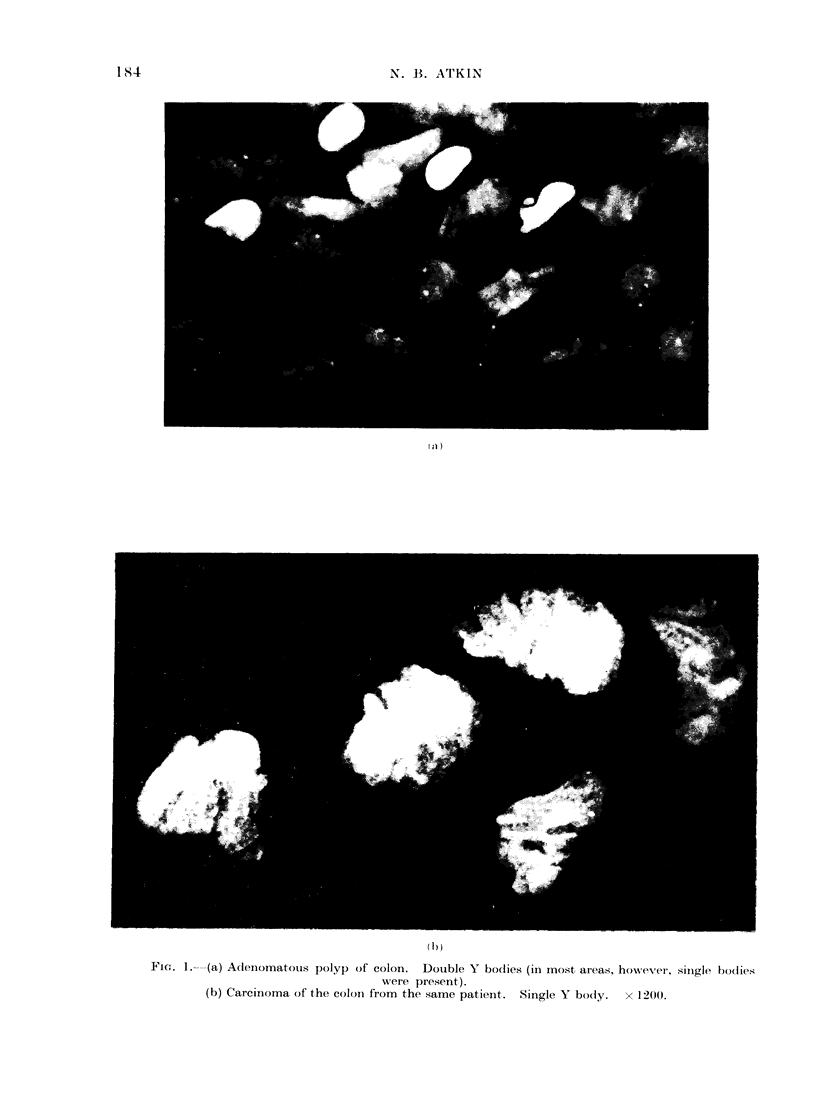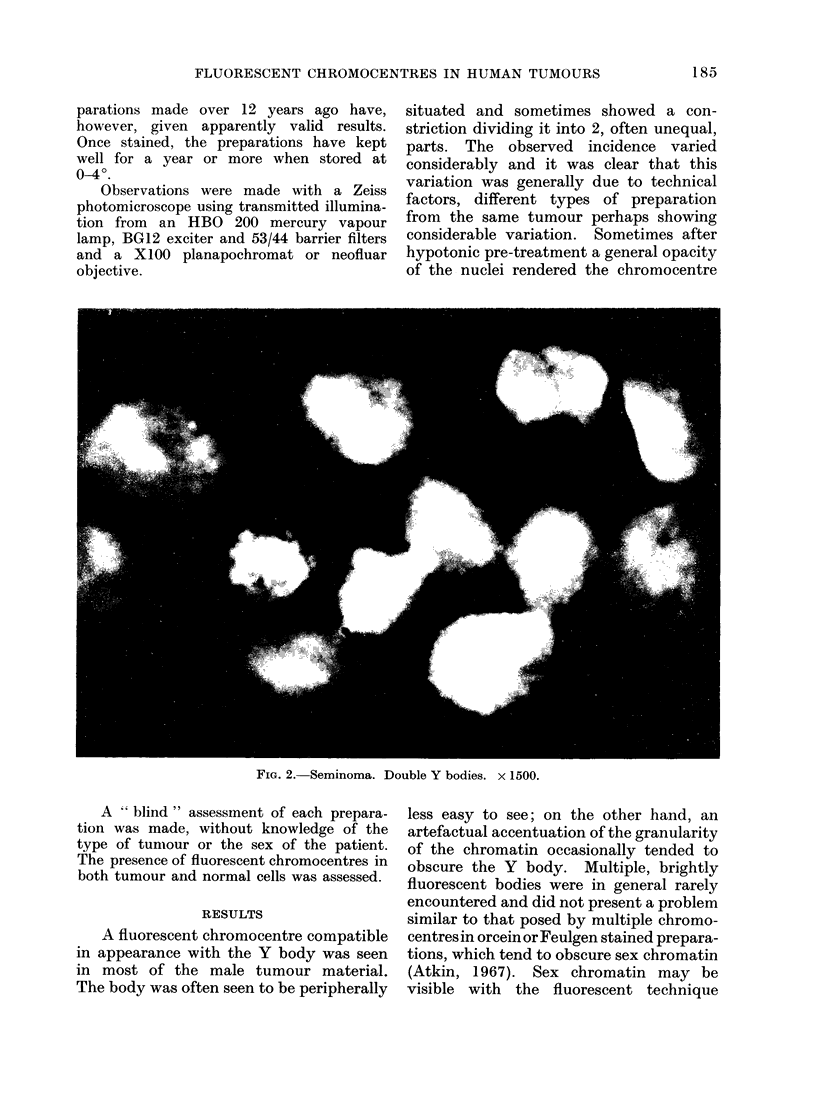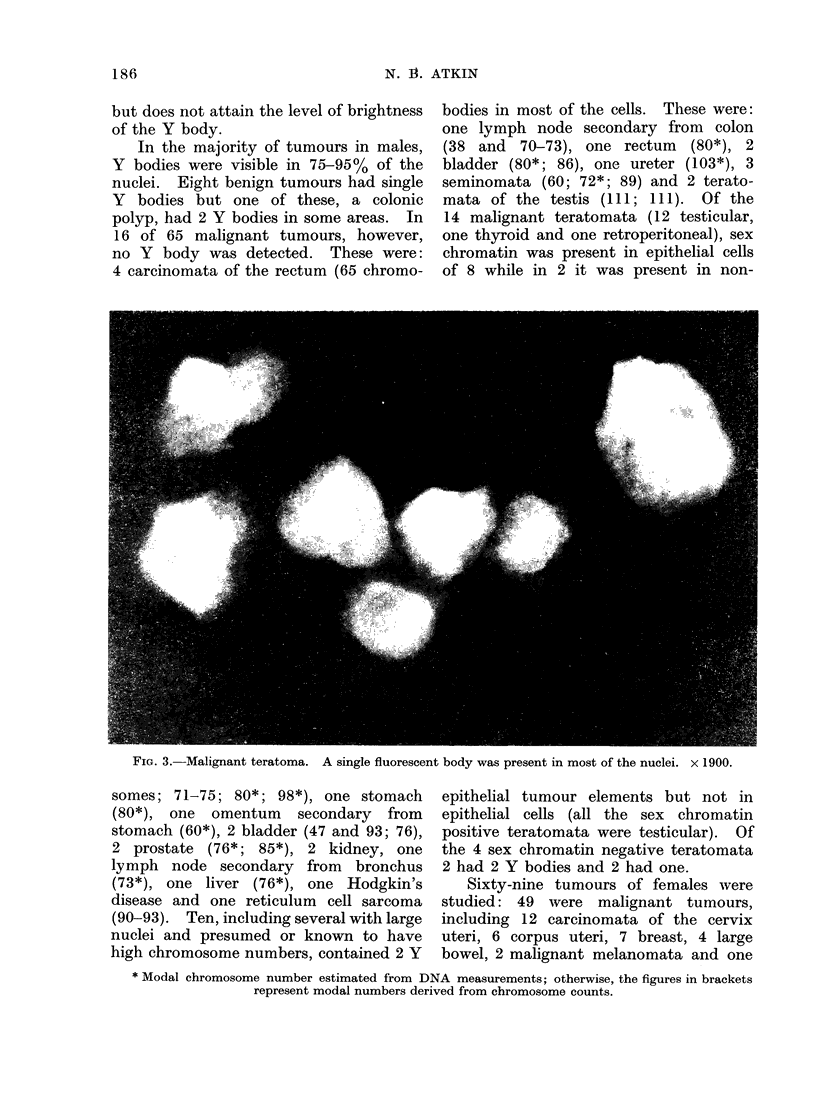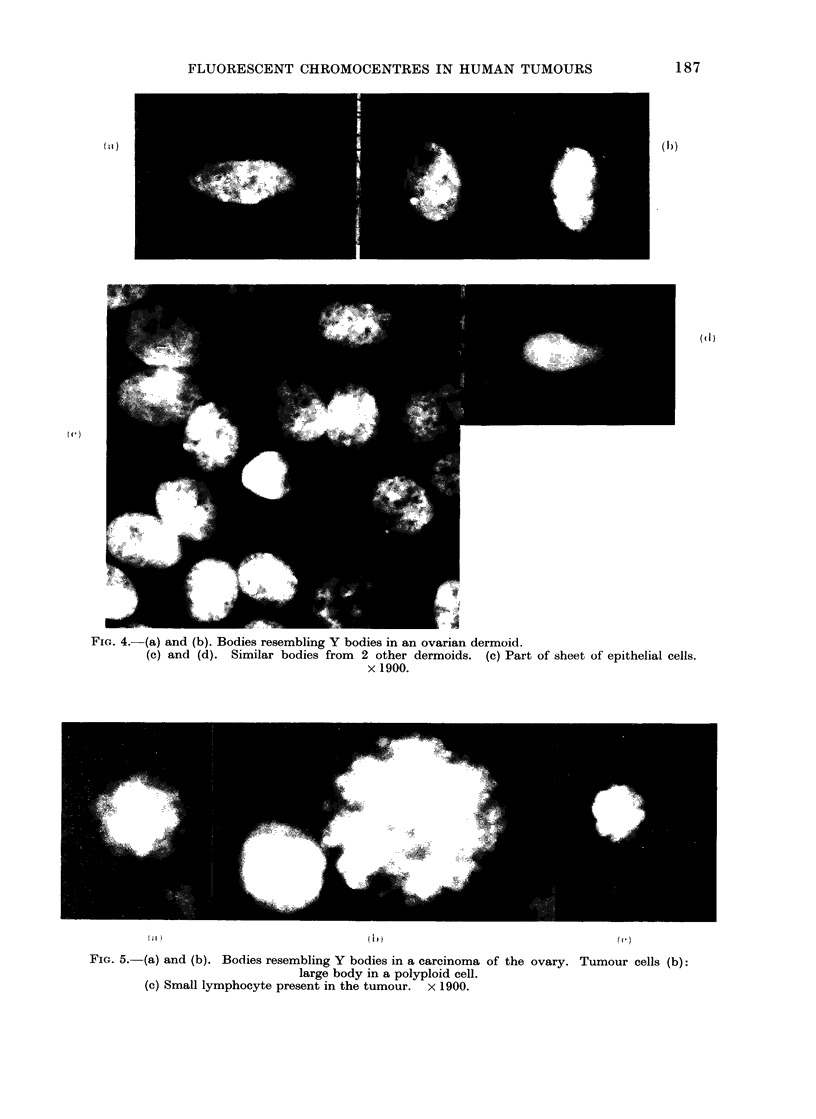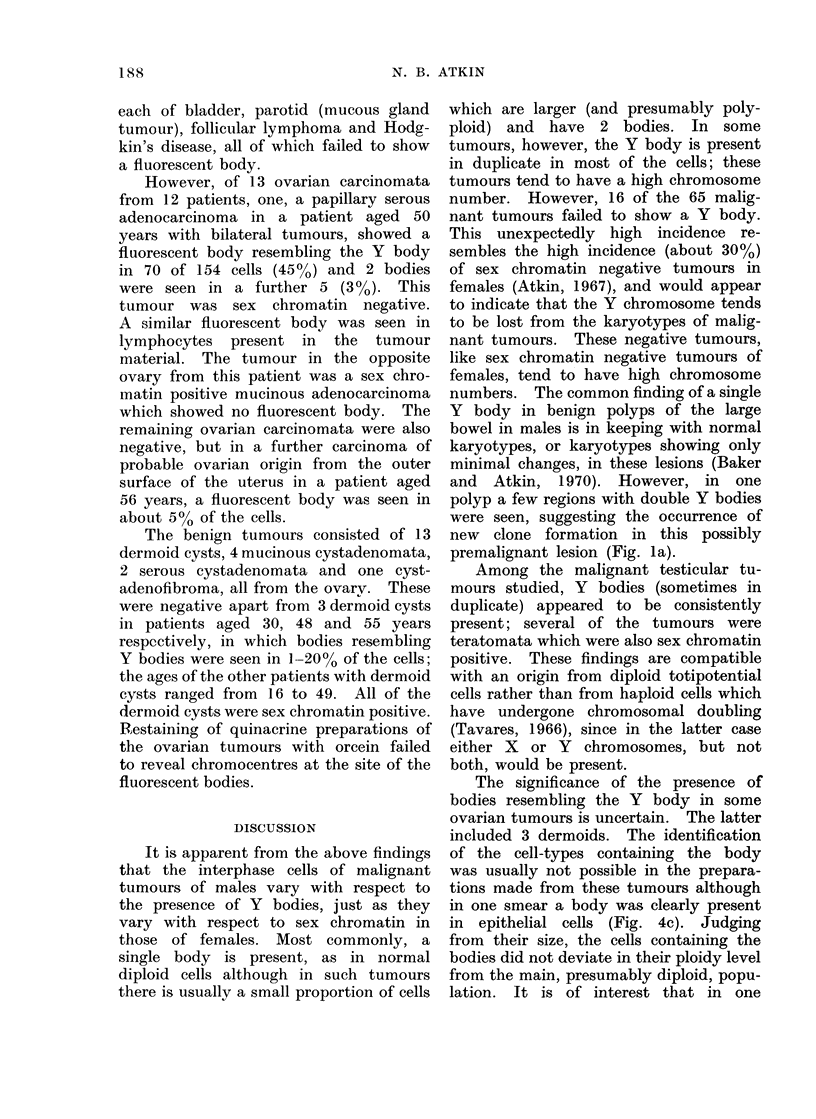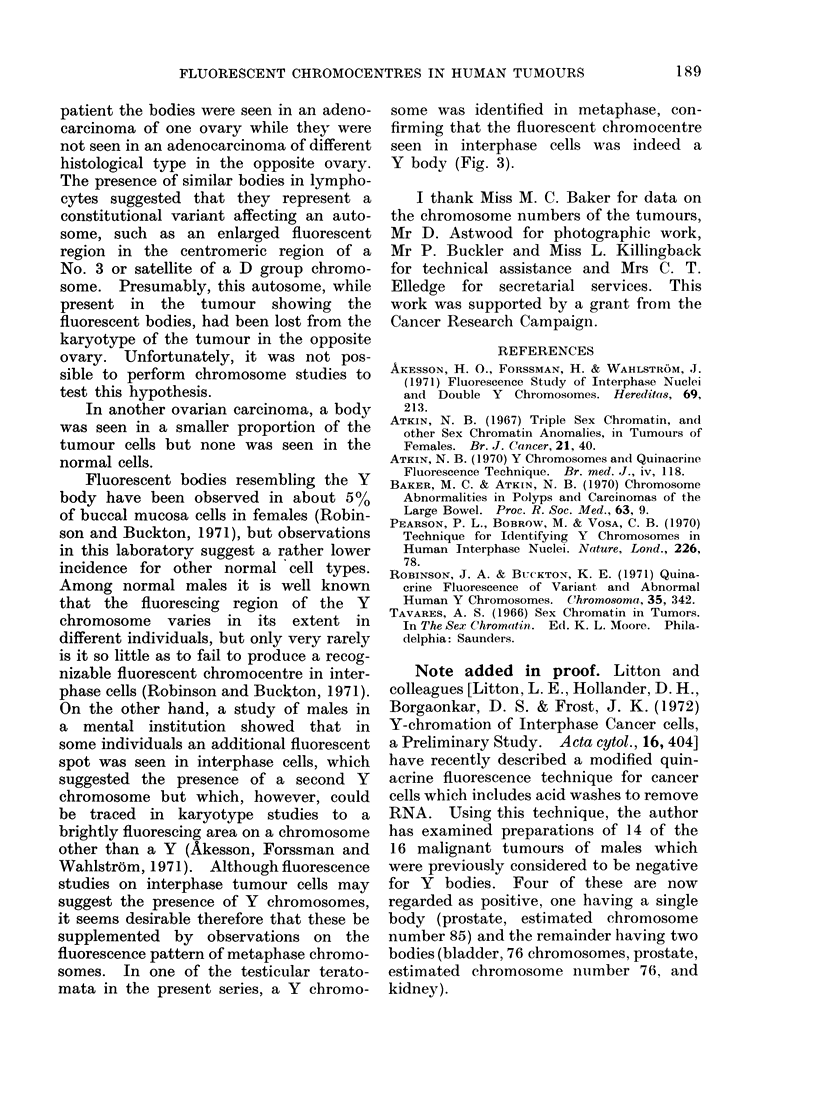# Y Bodies and Similar Fluorescent Chromocentres in Human Tumours Including Teratomata

**DOI:** 10.1038/bjc.1973.22

**Published:** 1973-02

**Authors:** N. B. Atkin

## Abstract

**Images:**


*Br. J. Cancer* (1973) **27**, 183.

### Y BODIES AND SIMILAR FLUORESCENT CHROMOCENTRES IN HUMAN TUMOURS INCLUDING TERATOMATA

N. B. ATKIN

*From the Department of Cancer Research, Mount Vernon Hospital, Northwood, Middlesex*

Received 21 July 1972. Accepted 16 October 1972

**Summary.—The presence of Y bodies and similar fluorescent chromocentres in the interphase cells of 73 benign and malignant neoplastic lesions of males, and 69 of females, has been assessed in preparations stained with quinacrine dihydrochloride. In the male, Y bodies were usually present, although none was seen in 16 of the 65 malignant tumours. Sometimes the Y body was present in duplicate, for example, in some regions of a benign polyp of the colon and generally in 10 of the malignant tumours. The series includes 5 seminomata and 12 malignant testicular teratomata, all of which were positive for Y bodies.**

**The tumours of females failed to show similar bodies, with 5 exceptions: one of 13 carcinomata of the ovary showed a body resembling the Y body in about half the cells (however, a similar body was seen in lymphocytes from this case) while a further carcinoma of probable ovarian origin, and 3 of 13 ovarian dermoids, showed a similar body though in less than 20% of the cells.**

**Although quinacrine fluorescence studies on interphase tumour cells may be of value in suggesting the presence or absence of Y chromosomes, it is desirable that these studies be supplemented by the investigation of the fluorescence pattern of the metaphase chromosomes.**

THE quinacrine fluorescence technique enables the presence of Y chromosomes to be determined from observations on interphase cells (Pearson, Bobrow and Vosa, 1970) and thus promises to provide an additional tool in the study of the chromosome changes in human tumours. This paper describes the findings in a variety of tumours, including several testicular and other teratomata. The presence of " Y bodies " in interphase cells from a malignant testicular teratoma has previously been reported briefly (Atkin, 1970); a study of Y chromosomes is of obvious interest in teratomata of males in view of their uncertain histogenesis and, in particular, the presence of sex chromatin in many tumours.

### MATERIALS AND METHODS

Quinacrine dihydrochloride from several sources has proved equally suitable. Various modifications of the staining technique have been tried; satisfactory results have been obtained by staining for 8 min in a 0·5% solution of the quinacrine in distilled water, rinsing in tap water and mounting in sodium acetate buffer (pH 5·5) or in deionized water, with the addition of one or 2 drops of glycerol to retard drying. Of the types of preparation of solid tumour material that were available, those made for chromosome studies by the conventional air-drying method have usually proved satisfactory (minced tumour tissue was subjected to hypotonic saline and Colcemid pre-treatment before fixation in 1 : 3 acetic acid : methanol). Smears fixed in 95% alcohol or by freeze substitution were generally satisfactory, but squashes of solid material fixed in acetic alcohol usually showed some granulation of the nuclei, which tended to obscure the fluorescent Y body. Material stored for some years in a refrigerator or domestic deep freeze unit has usually proved satisfactory, although the fluorescence may be less bright. Pre-

(a)

(b)

FIG. 1.—(a) Adenomatous polyp of colon. Double Y bodies (in most areas, however, single bodies were present).

(b) Carcinoma of the colon from the same patient. Single Y body. ×1200.

parations made over 12 years ago have, however, given apparently valid results. Once stained, the preparations have kept well for a year or more when stored at 0–4°.

Observations were made with a Zeiss photomicroscope using transmitted illumination from an HBO 200 mercury vapour lamp, BG12 exciter and 53/44 barrier filters and a X100 planapochromat or neofluar objective.

FIG. 2.—Seminoma. Double Y bodies. × 1500.

A " blind " assessment of each preparation was made, without knowledge of the type of tumour or the sex of the patient. The presence of fluorescent chromocentres in both tumour and normal cells was assessed.

### RESULTS

A fluorescent chromocentre compatible in appearance with the Y body was seen in most of the male tumour material. The body was often seen to be peripherally situated and sometimes showed a constriction dividing it into 2, often unequal, parts. The observed incidence varied considerably and it was clear that this variation was generally due to technical factors, different types of preparation from the same tumour perhaps showing considerable variation. Sometimes after hypotonic pre-treatment a general opacity of the nuclei rendered the chromocentre less easy to see; on the other hand, an artefactual accentuation of the granularity of the chromatin occasionally tended to obscure the Y body. Multiple, brightly fluorescent bodies were in general rarely encountered and did not present a problem similar to that posed by multiple chromocentres in orcein or Feulgen stained preparations, which tend to obscure sex chromatin (Atkin, 1967). Sex chromatin may be visible with the fluorescent technique but does not attain the level of brightness of the Y body.

In the majority of tumours in males, Y bodies were visible in 75–95% of the nuclei. Eight benign tumours had single Y bodies but one of these, a colonic polyp, had 2 Y bodies in some areas. In 16 of 65 malignant tumours, however, no Y body was detected. These were: 4 carcinomata of the rectum (65 chromosomes; 71–75; 80*; 98*), one stomach (80*), one omentum secondary from stomach (60*), 2 bladder (47 and 93; 76), 2 prostate (76*; 85*), 2 kidney, one lymph node secondary from bronchus (73*), one liver (76*), one Hodgkin's disease and one reticulum cell sarcoma (90–93). Ten, including several with large nuclei and presumed or known to have high chromosome numbers, contained 2 Y bodies in most of the cells. These were: one lymph node secondary from colon (38 and 70–73), one rectum (80*), 2 bladder (80*; 86), one ureter (103*), 3 seminomata (60; 72*; 89) and 2 teratomata of the testis (111; 111). Of the 14 malignant teratomata (12 testicular, one thyroid and one retroperitoneal), sex chromatin was present in epithelial cells of 8 while in 2 it was present in non-epithelial tumour elements but not in epithelial cells (all the sex chromatin positive teratomata were testicular). Of the 4 sex chromatin negative teratomata 2 had 2 Y bodies and 2 had one.

FIG. 3.—Malignant teratoma. A single fluorescent body was present in most of the nuclei. × 1900.

Sixty-nine tumours of females were studied: 49 were malignant tumours, including 12 carcinomata of the cervix uteri, 6 corpus uteri, 7 breast, 4 large bowel, 2 malignant melanomata and one

* Modal chromosome number estimated from DNA measurements; otherwise, the figures in brackets represent modal numbers derived from chromosome counts.

FIG. 4.—(a) and (b). Bodies resembling Y bodies in an ovarian dermoid.
(c) and (d). Similar bodies from 2 other dermoids. (c) Part of sheet of epithelial cells.
× 1900.

FIG. 5.—(a) and (b). Bodies resembling Y bodies in a carcinoma of the ovary. Tumour cells (b): large body in a polyploid cell.
(c) Small lymphocyte present in the tumour. × 1900.

each of bladder, parotid (mucous gland tumour), follicular lymphoma and Hodgkin's disease, all of which failed to show a fluorescent body.

However, of 13 ovarian carcinomata from 12 patients, one, a papillary serous adenocarcinoma in a patient aged 50 years with bilateral tumours, showed a fluorescent body resembling the Y body in 70 of 154 cells (45%) and 2 bodies were seen in a further 5 (3%). This tumour was sex chromatin negative. A similar fluorescent body was seen in lymphocytes present in the tumour material. The tumour in the opposite ovary from this patient was a sex chromatin positive mucinous adenocarcinoma which showed no fluorescent body. The remaining ovarian carcinomata were also negative, but in a further carcinoma of probable ovarian origin from the outer surface of the uterus in a patient aged 56 years, a fluorescent body was seen in about 5% of the cells.

The benign tumours consisted of 13 dermoid cysts, 4 mucinous cystadenomata, 2 serous cystadenomata and one cystadenofibroma, all from the ovary. These were negative apart from 3 dermoid cysts in patients aged 30, 48 and 55 years respectively, in which bodies resembling Y bodies were seen in 1–20% of the cells; the ages of the other patients with dermoid cysts ranged from 16 to 49. All of the dermoid cysts were sex chromatin positive. Restaining of quinacrine preparations of the ovarian tumours with orcein failed to reveal chromocentres at the site of the fluorescent bodies.

### DISCUSSION

It is apparent from the above findings that the interphase cells of malignant tumours of males vary with respect to the presence of Y bodies, just as they vary with respect to sex chromatin in those of females. Most commonly, a single body is present, as in normal diploid cells although in such tumours there is usually a small proportion of cells which are larger (and presumably polyploid) and have 2 bodies. In some tumours, however, the Y body is present in duplicate in most of the cells; these tumours tend to have a high chromosome number. However, 16 of the 65 malignant tumours failed to show a Y body. This unexpectedly high incidence resembles the high incidence (about 30%) of sex chromatin negative tumours in females (Atkin, 1967), and would appear to indicate that the Y chromosome tends to be lost from the karyotypes of malignant tumours. These negative tumours, like sex chromatin negative tumours of females, tend to have high chromosome numbers. The common finding of a single Y body in benign polyps of the large bowel in males is in keeping with normal karyotypes, or karyotypes showing only minimal changes, in these lesions (Baker and Atkin, 1970). However, in one polyp a few regions with double Y bodies were seen, suggesting the occurrence of new clone formation in this possibly premalignant lesion (Fig. 1a).

Among the malignant testicular tumours studied, Y bodies (sometimes in duplicate) appeared to be consistently present; several of the tumours were teratomata which were also sex chromatin positive. These findings are compatible with an origin from diploid totipotential cells rather than from haploid cells which have undergone chromosomal doubling (Tavares, 1966), since in the latter case either X or Y chromosomes, but not both, would be present.

The significance of the presence of bodies resembling the Y body in some ovarian tumours is uncertain. The latter included 3 dermoids. The identification of the cell-types containing the body was usually not possible in the preparations made from these tumours although in one smear a body was clearly present in epithelial cells (Fig. 4c). Judging from their size, the cells containing the bodies did not deviate in their ploidy level from the main, presumably diploid, population. It is of interest that in one patient the bodies were seen in an adenocarcinoma of one ovary while they were not seen in an adenocarcinoma of different histological type in the opposite ovary. The presence of similar bodies in lymphocytes suggested that they represent a constitutional variant affecting an autosome, such as an enlarged fluorescent region in the centromeric region of a No. 3 or satellite of a D group chromosome. Presumably, this autosome, while present in the tumour showing the fluorescent bodies, had been lost from the karyotype of the tumour in the opposite ovary. Unfortunately, it was not possible to perform chromosome studies to test this hypothesis.

In another ovarian carcinoma, a body was seen in a smaller proportion of the tumour cells but none was seen in the normal cells.

Fluorescent bodies resembling the Y body have been observed in about 5% of buccal mucosa cells in females (Robinson and Buckton, 1971), but observations in this laboratory suggest a rather lower incidence for other normal cell types. Among normal males it is well known that the fluorescing region of the Y chromosome varies in its extent in different individuals, but only very rarely is it so little as to fail to produce a recognizable fluorescent chromocentre in interphase cells (Robinson and Buckton, 1971). On the other hand, a study of males in a mental institution showed that in some individuals an additional fluorescent spot was seen in interphase cells, which suggested the presence of a second Y chromosome but which, however, could be traced in karyotype studies to a brightly fluorescing area on a chromosome other than a Y (Åkesson, Forssman and Wahlström, 1971). Although fluorescence studies on interphase tumour cells may suggest the presence of Y chromosomes, it seems desirable therefore that these be supplemented by observations on the fluorescence pattern of metaphase chromosomes. In one of the testicular teratomata in the present series, a Y chromosome was identified in metaphase, confirming that the fluorescent chromocentre seen in interphase cells was indeed a Y body (Fig. 3).

I thank Miss M. C. Baker for data on the chromosome numbers of the tumours, Mr D. Astwood for photographic work, Mr P. Buckler and Miss L. Killingback for technical assistance and Mrs C. T. Elledge for secretarial services. This work was supported by a grant from the Cancer Research Campaign.

REFERENCES

Åkesson, H. O., Forssman, H. & Wahlström, J. (1971) Fluorescence Study of Interphase Nuclei and Double Y Chromosomes. *Hereditas*, **69**, 213.

Atkin, N. B. (1967) Triple Sex Chromatin, and other Sex Chromatin Anomalies, in Tumours of Females. *Br. J. Cancer*, **21**, 40.

Atkin, N. B. (1970) Y Chromosomes and Quinacrine Fluorescence Technique. *Br. med. J.*, iv, 118.

Baker, M. C. & Atkin, N. B. (1970) Chromosome Abnormalities in Polyps and Carcinomas of the Large Bowel. *Proc. R. Soc. Med.*, **63**, 9.

Pearson, P. L., Bobrow, M. & Vosa, C. B. (1970) Technique for Identifying Y Chromosomes in Human Interphase Nuclei. *Nature, Lond.*, **226**, 78.

Robinson, J. A. & Buckton, K. E. (1971) Quinacrine Fluorescence of Variant and Abnormal Human Y Chromosomes. *Chromosoma*, **35**, 342.

Tavares, A. S. (1966) Sex Chromatin in Tumors. In *The Sex Chromatin*. Ed. K. L. Moore. Philadelphia: Saunders.

**Note added in proof.** Litton and colleagues [Litton, L. E., Hollander, D. H., Borgaonkar, D. S. & Frost, J. K. (1972) Y-chromation of Interphase Cancer cells, a Preliminary Study. *Acta cytol.*, **16**, 404] have recently described a modified quinacrine fluorescence technique for cancer cells which includes acid washes to remove RNA. Using this technique, the author has examined preparations of 14 of the 16 malignant tumours of males which were previously considered to be negative for Y bodies. Four of these are now regarded as positive, one having a single body (prostate, estimated chromosome number 85) and the remainder having two bodies (bladder, 76 chromosomes, prostate, estimated chromosome number 76, and kidney).